# Investigation on the influence of co-sensitization on semi-transparent DSSCs fabricated using NIR-sensitive squaraine dyes and visible dyes

**DOI:** 10.1038/s41598-025-90337-0

**Published:** 2025-02-25

**Authors:** Nur Izyan, Adam Glinka, Safalmani Pradhan, Chinmai Mysorekar, Shyam Sudhir Pandey, Marcin Ziółek

**Affiliations:** 1 Graduate School of Life Science and Systems Engineering, Kyushu Institute of Technology, 2,4-Hibikino, Wakamatsu, Kitakyushu 808-0196 Japan; 2 Faculty of Physics and Astronomy, Adam Mickiewicz University, 2 Uniwersytetu Poznańskiego, 61-614 Poznań, Poland

**Keywords:** Dye sensitized solar cells, Co-sensitization, Squaraine dyes, Transient absorption spectroscopy, Semi-transparent cells, Electron transfer, Solar cells, Solar cells, Solar cells

## Abstract

Two squaraine dyes (“blue colored” SQ258 and “green colored” SQ259) active in the near-infrared spectral range were co-sensitized with popular commercial dyes (D35 and N719) and their photophysical characterizations were performed using stationary and time-resolved spectroscopic techniques. The dye mixtures, D35:SQ258 and N719:SQ259 demonstrated a better photovoltaic performance when compared with their individual photovoltaic efficiencies. The studied different DSSC configurations using squaraine dyes and mixtures in the devices with thin TiO_2_ layer show the potential application in multi-color semi-transparent cells. The D35:SQ258 system was also evaluated in the presence of cobalt-based redox shuttle, revealing improved performance compared to I^−^/I_3_^−^. The stationary, transient absorption and external quantum efficiency spectra indicated the squaraine dye de-aggregating function of D35 and N719 dyes. The fast internal conversion of the excited squaraine dyes which competes with too slow electron injection was observed as the main reason for the limited charge separation efficiency in the studied cells of pure squaraine dyes. However, as a result of the synergetic effects of the co-sensitized dyes, the improvement of the charge separation efficiency was revealed. Finally, transient absorption studies revealed that upon exclusive excitation of squaraine dyes an ultrafast energy transfer between the squaraine monomers and their H-aggregates takes place, and a Stark shift of absorption band of co-sensitized dyes can be observed.

**Supplementary Information:**

The online version contains supplementary material available at 10.1038/s41598-025-90337-0.

## Introduction

The advancement of technology and the modernization of human life has led to a huge increase in energy consumption, thereby leading to an enormous rise in energy demand. According to a report by International Energy Agency, global demand for electricity has been forecasted to grow at a rapid rate over the next three years, increasing by an average of 3.4% annually through 2026^1^. Since most of the energy that is consumed across the world comes from fossil fuels, it is the production of this energy that is responsible for 87% of greenhouse gas emissions, thereby resulting in global warming and climate change^2^. The seriousness of this matter has drawn global attention prompting scientists to find a sustainable energy sources as one of the plausible solutions to tackle the problem. Since solar energy is one of the most widely available renewable energy sources, its conversion into readily consumable electrical energy with the help of photovoltaic technology can fulfill the huge energy demand. Part of the growing power deficit could be also satisfied where it is created by application of photovoltaic modules in small electronic devices making them independent of toxic disposable batteries or accumulators. In this context, dye-sensitized solar cells (DSSCs), a type of third-generation solar cells provides not only a cost-effective solution, based on relatively environmentally friendly materials, but also a platform for tailoring multi-color and semi-transparent photovoltaic devices attractive as aesthetic product integrated components.

Significant progress has been made ever since DSSCs was first reported in 1991 by Graetzel and O’Regan where they demonstrated the photoconversion efficiency (PCE) of 7.9%, utilizing ruthenium complex based dye^3^. For decades research efforts has been focusing on improving the efficiency of DSSCs through the improvements of semiconductor materials, redox electrolytes, counter electrodes, and sensitizers^4,5^. As a result highest PCE of > 15% was recently achieved^6^. Ruthenium coordination complex based dyes have been proven to be an excellent sensitizer due to their stability, and high PCE^7^. However, the high cost and scarcity of these dyes, coupled with their complicated synthesis and purification methods, have diverted researchers’ attention to metal-free organic sensitizers. The metal free sensitizer, however, must exhibit panchromatic photon harvesting to function as an efficient sensitizer in a DSSC. In order to extend the range of effective light harvesting, dyes which efficiently absorb near infrared (NIR) photons (photons beyond 700 nm region) were introduced^8,9^.

The NIR dyes (including the squaraine dyes studied in this work) have also significant potential for application in transparent or semi-transparent DSSCs^10–13^ due to low absorption in the visible region, but also because they provide blue-green range colors which are very rear among the palate of DSSC dyes. However, due to the lower energy bandgap of these dyes, special attention is required to address potential issues with energy level misalignment and aggregation-related phenomena. These factors can limit the charge separation efficiency and thus the PCE of the devices incorporating such dyes^14–17^. In this context, fundamental studies revealing fast dynamics of charge separation are particularly crucial.

It should be noted that almost all of the current top efficient DSSC (PCE > 13%) incorporate at least two co-sensitized dyes^6,18–21^. However, there are only few studies that examine the photophysical interactions of dyes in co-sensitized systems in sub-picosecond to nanosecond timescale^22,23^. Therefore, the direct mechanism causing such high PCEs is still underexplored, in particular the way co-sensitization of the two dyes affects the dynamics of charge separation.

The effect of co-sensitization on dye aggregations in DSSC has been reported before^24–27^. However, to the best of our knowledge, it has been shown quite rarely for squaraine systems^28,29^, and has never been investigated or confirmed through the time-resolved spectroscopic studies. Moreover, so far squaraine dyes are the systems mainly studied in iodide electrolyte with only a few reports for alternative redox shuttles, such as cobalt-based ones^17,30,31^.

Therefore, in this work we focused on the mechanism of electron injection in NIR squaraine dyes, the effects of the co-sensitization and dye aggregation. We use two novel NIR-sensitive unsymmetrical squaraine dyes, SQ258 and SQ259 (Scheme S1, Supporting Information) that were synthesized and reported previously^32^. In the current work the above dyes were studied by time-resolved spectroscopic techniques in order to understand the electron transfer processes in DSSCs and improve their charge separation efficiency by the electrolyte modification. Moreover, our study also deals with the fabrication of DSSCs by co-sensitizing SQ258 and SQ259 with two commercially available visible light-sensitive dyes D35^33^ and N719^34^ (Scheme S1). It is shown that the co-sensitization of the visible and the NIR dye led to a complementary photon harvesting in the entire 400–750 nm region, i.e., panchromatic photon harvesting. Furthermore, the effect of bulky moieties of D35 dye, which suppresses the conduction band electron recapture was assessed in the co-sensitized system using [Co(bpy)_3_]^2+/3+^ redox couple. The study revealed that the passivating effect was maintained even for low (1:5) D35:SQ258 dye ratios, and it also enhanced the possibility that the squaraine dye could be used in effective co-sensitized systems mediated by Co-based redox shuttle.

The present work demonstrates a significant improvement in PCE for squaraine-based DSSCs. It also explores several important phenomena, such as mutual interactions within co-sensitized systems, the effects of aggregation and new findings related to Stark shift effect^35^. Additionally, it examines the influence of different electrolyte compositions and the charge dynamics in aggregates and co-sensitized systems. Understanding these interactions is essential, not only to elucidate the achievement of high PCE in the state-of-the-art DSSCs, but also to guide further innovations and improvements in the solar cell technology.

## Materials and methods

The photoanodes were prepared by screen-printing of a single TiO_2_ layer (GreatCell 30 NRD-T paste using 250 mesh count, Sefar). Before screen-printing of the TiO_2_, the FTO substrates were subjected to TiCl_4_ treatment by dipping in 40 mM aqueous TiCl_4_ solution at 70 °C for 30 min. The screen printing of TiO_2_ was followed again by TiCl_4_ treatment under the same conditions. The substrates were subjected to annealing after both pre- and post-TiCl_4_ treatment. The thickness of the mesoporous TiO_2_ layer was measured to be ~ 3 μm. Relatively thin TiO_2_ layers were used (in contrast to that in the most efficient DSSC) because of the requirement for fabricating semi-transparent cells. Such cells also enable for carrying out absorption measurements (both stationary so that the dyes absorption band maxima can be clearly seen—section “Stationary absorption”, and time-resolved to provide enough probing light in transient absorption measurements—section “Transient absorption”). The photoanodes were immersed in solutions of the respective dyes (0.2 mM of both SQ258 and SQ259) and their mixtures with D35 or N719 (at several different proportions from 2:1 to 1:7) in ethanol. Chenodeoxycholic acid (CDCA), an anti-aggregation agent was also used for selected cells at the concentrations optimized before (30 mM for SQ258 and 20 mM for SQ259)^32^. The counter electrodes were prepared by depositing a layer of activated platinum on a FTO glass. The photoanodes and counter electrodes were attached together through a polymer seal (25 μm Surlyn) with the conducting surfaces of both the electrodes facing each other. Following this, the devices were filled with electrolyte through 1 mm holes in the counter electrode and sealed with a cover glass on the top. Three acetonitrile-based electrolytes were used. The first type of electrolyte was the same as the one used in the previous work^32^, which consisted of iodine (I_2_) (0.05 M), lithium iodide (LiI) (0.1 M), t-butyl pyridine (TBP, 0.5 M), and 1,2-dimethyl-3-propyl imidazolium iodide (0.6 M). The second type of electrolyte was prepared without TBP (aimed at shifting TiO_2_ conduction band towards more positive potentials) and contained 0.05 M I_2_ and 0.6 M LiI. The last type comprised of 0.25 M [Co(bpy)_3_](B(CN)_4_)_2_, (bpy = 2,2′-bipyridine) 0.035 M [Co(bpy)_3_](B(CN)_4_)_3_, 0.1 M LiTFSI (TFSI = bis(trifluoromethane)sulfonamide) and 0.5 M TBP.

Stationary absorption spectra were measured using a UV-VIS-NIR JASCO V-770 spectrophotometer equipped with a 150 mm integrating sphere (LN-925). The samples were placed in front of the integrating sphere to detect both transmitted and scattered light. Current–voltage (I-V) measurements and incident photon to current conversion efficiency (IPCE) spectra for the fabricated DSSCs were recorded using a potentiostat (model M101, Autolab) coupled to a photoelectric spectrometer, equipped with a solar simulator (Instytut Fotonowy, Poland). The sunlight conditions were provided by a Xe lamp with AM 1.5G spectral filter and irradiance adjusted to 100 mW cm^2^ using a calibrated cell (15151, ABET).

Transient absorption (TA) spectroscopic measurements of the fully operational devices was performed using femtosecond transient absorption spectrometer (Helios, Ultrafast Systems) described before in detail^36^. The excitation wavelength was set at 475, 620 or725 nm and the pulse energy was varied between 6 and 260 nJ. The detection was performed in the range between 480 nm and 830 nm. Time window and resolution of the measurements were respectively limited by the range of delay line (3 ns) and instrument response function (IRF) duration (~ 0.2 ps). The time and wavelength dependent transient absorption data was processed using Surface Explorer software provided by Ultrafast Systems. The artifact originating from group velocity dispersion (so called chirp) was corrected^37^. The global analysis using this software also allowed fitting a multi-exponential function (convoluted with response function) to the kinetic vectors of a selected number of singular values. Then, the characteristic time constants and the wavelength‐dependent amplitudes associated with them were obtained.

## Results and discussion

### Stationary absorption

**Fig. 1:**
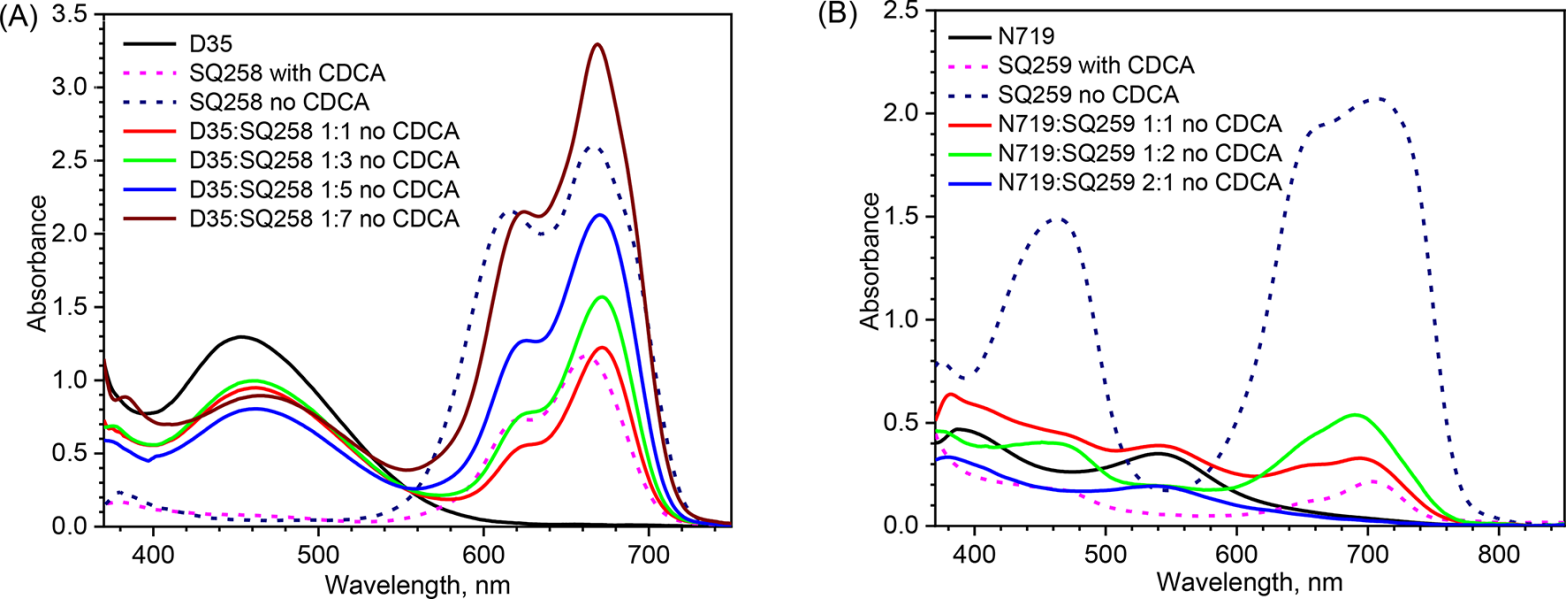
Stationary absorption spectra of the photoanodes sensitized with different dye configurations for SQ258 (**A**) and SQ259 (**B**). Pure TiO_2_ contribution was subtracted from all spectra.

Stationary absorption spectra of different photoanodes are shown in Fig. 1. It is observed that the squaraine dyes have broad absorption bands at the long wavelengths range, from 550 nm to 700 nm for SQ258 (Fig. 1A), and from 550 nm to 750 nm for SQ259 (Fig. 1B), respectively. For SQ259, another absorption band of relatively lower intensity is observed at the shorter wavelength region (400–500 nm). As reported before^32^, the absorption spectra of both SQ258 and SQ259 after their adsorption on the photoanodes, their solid-state absorption spectra are much broader than those in solution (see also Fig. S1). This broadening of the solid-state absorption spectra has been attributed to severe aggregation of the dye molecules on the surface of mesoporous TiO_2_. The intensification of the vibronic shoulder at the blue (shorter wavelength) region of the spectrum is particularly attributed to the formation of H-aggregates^32,38^. The spectral broadening towards the longer wavelength regions has been attributed to the formation of J-aggregates^39,40^. Since the formation of the dye aggregates typically lowers the photovoltaic performance of a DSSC, relatively high amounts of CDCA as a de-aggregation agent has to be added (generally in concentrations that is 100–150 times higher than the concentration of the squaraine dyes in sensitizing solutions)^32,41^. As reported before, it can be seen that the absorption spectrum of both the squaraine dyes, SQ258 and SQ259 exhibited relatively sharp and narrow absorption bands after the addition of CDCA (with less intense vibronic shoulder). However, Fig. 1 also shows that the maximum absorbance of the dyes containing CDCA is much weaker (about 3–5 times weaker) than those without CDCA, which might influence the photovoltaic performance of the DSSCs. Given the preparation of photoanodes with relatively thin TiO_2_ layers in the present work, the use of CDCA might therefore more severely lower the number of absorbed photons (N_ph_) from 1Sun spectrum than for the thick cells optimized for high PCE. The measured absorption spectra for all the photoanodes and the data for 1Sun photon flux were used to estimate N_ph_ which will be used in the next section to calculate the relative photocurrent (total APCE), the parameter which is in principle independent from the cell thickness.

Next, the commercially available dyes, D35 and N719, which are reported to be quite efficient in iodide-based electrolytes, were used as co-sensitizer on the mesoporous TiO_2_ along with NIR dyes, SQ258 and SQ259, respectively. The principal reason for their selection was their strong absorption of light in the visible region which would effectively complement the absorption of light in the NIR region by SQ258 and SQ259 (Fig. 1). In contrast to sequential adsorption^42^, co-sensitization of mesoporous TiO_2_ was accomplished by single immersion in dye cocktails at different molar ratios. The absorption spectra in Fig. 1 clearly show that the use of visible-sensitive dyes does not only result in the complementary photon harvesting (in the visible region, where generally squaraine dyes do not exhibit photon harvesting) but D35 and N719 dyes also function as an anti-aggregation agent (considerably reducing the formation of squaraine dye aggregates). Thus, they play a role that is similar to that of CDCA in DSSC. This observation can be proven by the fact that the increase in the molar ratio of D35 in the D35:SQ258 dye cocktail (Fig. 1A) consecutively from 1:7, 1:5, 1:3 up to 1:1 molar ratio (without CDCA) led to corresponding reduction in the intensity of the vibronic shoulder of SQ258. In fact, the intensity of vibronic shoulder at ~ 610 nm for 1:1 (D35:SQ258) dye cocktail and SQ258 with CDCA with respect to the main absorption peak at ~ 660 nm is quite similar meaning that the dye D35 successfully prevented squaraine dye aggregate formation. Similar conclusions can be drawn for N719:SQ259 mixture for which the squaraine band is red-shifted (vibronic shoulder at ~ 660 nm and main peak at ~ 700 nm, Fig. 1B). The IPCE spectra and the transient absorption data presented in the next sections will further confirm that the visible dyes used as a co-sensitizer aid in preventing aggregate formation. It is noteworthy mentioning that the use of visible dyes as co-sensitizers with squaraine dyes offers an advantage in enhancing the photovoltaic performance of solar cell. This dual benefit arises from the ability of visible dyes to achieve panchromatic photon harvesting while simultaneously preventing the aggregation of squaraine dyes. In comparison, CDCA also mitigates aggregation but compromises light absorption. This limitation occurs because CDCA competes with squaraine dyes for adsorption sites on mesoporous TiO_2_, ultimately reducing the loading of squaraine dyes and diminishing the overall light-harvesting efficiency of the system. The studied configurations exhibit varying absorption levels across different regions of the visible spectrum. However, in each case, there is at least one part of the spectrum where the absorbance is relatively low (Fig. S2). As will be shown below, this characteristic facilitates the development of relatively efficient, colorful, semi-transparent devices.

### Photovoltaic performance

The DSSCs thus fabricated using the dye cocktails were subjected to photovoltaic characterization under 1 Sun illumination. Typically for each configuration at least 3 cells were prepared. The results for the best cells for each series are presented in Table 1. The relative error (standard deviation divided by the mean value) of photovoltaic parameters varies from 4 to 10%, as indicated in Table S1 (in the Supporting Information).

It should be noted that relatively low PCE values are due to the thinner TiO_2_ layer used in our cells (with respect to the top efficiency DSSC devices) because of the requirement of maintaining the transparency of the cells to enable transient absorption studies. Moreover, photos in Fig. S1C, D and transmittance spectra of the full cells (Fig. S2) confirm the semi-transparency of the cells in our study, which, together with the large variety of colors available for different configurations (orange, green, blue) makes them an interesting system of decorative applications. Besides the basic photovoltaic parameters, such as open circuit voltage (V_OC_), short circuit current density (J_SC_) and fill factor (FF) the tables show the number of absorbed photons from 1 Sun (N_ph_) and the relative photocurrent, also known as total APCE and defined as J_SC_/(e × N_ph_), where the e is elementary charge^43^. The latter parameter is especially suitable for the studies of thinner solar cells because it enables to compare the total charge separation efficiency (at short circuit conditions) of different system irrespective of the light absorption and can be used to predict the photocurrent in the optimized, thicker cells with higher N_ph_.

**Table 1:** Photovoltaic parameters of the best cells of each series (for the electrolytes: “I” indicates iodide electrolyte with TBP, “I no T”—iodide electrolyte without TBP, and “Co”—cobalt-based electrolyte with TBP).

Sample abbreviation	Dyes and mixture ratios	CDCA	Electrolyte	V_OC_ [V]	FF	J_SC_ [mA/cm^2^]	PCE [%]	*N*_Ph_ [10^20^ s^−1^ m^−2^]	Total APCE
D	D35	No	I	0.81	0.58	4.18	1.96	4.71	0.55
S8_CDCA	SQ258	Yes	I	0.60	0.76	2.38	1.08	4.61	0.32
S8	SQ258	No	I	0.54	0.67	4.20	1.53	7.87	0.33
D:S8_1	D35:SQ258 1:1	No	I	0.69	0.71	3.87	1.90	7.81	0.31
D:S8_3	D35:SQ258 1:3	No	I	0.63	0.67	5.57	2.36	9.37	0.37
**D:S8_5**	**D35:SQ258 1:5**	**No**	I	**0.67**	**0.67**	**7.39**	**3.31**	**9.85**	**0.47**
D:S8_7	D35:SQ258 1:7	No	I	0.64	0.65	6.72	2.78	11.42	0.37
**D:S8_1_CDCA**	**D35:SQ258 1:1**	**Yes**	I	**0.70**	**0.70**	**5.54**	**2.71**	**5.13**	**0.67**
D:S8_3_CDCA	D35:SQ258 1:1	Yes	I	0.69	0.75	4.49	2.44	4.83	0.61
N	N719	No	I	0.70	0.76	3.80	2.01	6.78	0.35
S9_CDCA	SQ259	Yes	I	0.63	0.69	3.45	1.50	4.83	0.45
S9	SQ259	No	I	0.48	0.60	3.99	1.14	11.51	0.22
**N:S9_1**	**N719:SQ259 1:1**	**No**	**I**	**0.60**	**0.73**	**4.45**	**1.95**	**7.76**	**0.36**
N:S9_2	N719:SQ259 1:2	No	I	0.57	0.76	3.45	1.51	7.48	0.29
D_Co	D35	No	Co	0.98	0.55	4.75	2.57	5.10	0.58
D:S8_Co	SQ258	No	Co	0.70	0.68	2.99	1.40	8.42	0.22
**D:S8_5_Co**	**D35:SQ258 1:5**	**No**	**Co**	**0.78**	**0.71**	**6.35**	**3.51**	**9.90**	**0.40**
S9_Co	SQ259	No	Co	0.67	0.73	1.59	0.77	10.12	0.10
D_noT	D35	No	I no T	0.61	0.47	5.15	1.47	4.81	0.67
S8_CDCA_noT	SQ258	Yes	I no T	0.44	0.61	1.96	0.53	1.96	0.63
S8_noT	SQ258	No	I no T	0.50	0.58	7.56	2.21	7.84	0.60
D:S8_1_noT	D35:SQ258 1:1	No	I no T	0.56	0.46	8.85	2.29	8.55	0.65
D:S8_3_noT	D35:SQ258 1:3	No	I no T	0.54	0.51	7.70	2.13	9.35	0.51
**D:S8_5_noT**	**D35:SQ258 1:5**	**No**	**I no T**	**0.51**	**0.50**	**10.84**	**2.75**	**9.21**	**0.74**
D:S8_7_noT	D35:SQ258 1:7	No	I no T	0.43	0.43	10.45	1.94	11.59	0.56
N_noT	N719	No	I no T	0.50	0.61	6.15	1.90	4.19	0.92
S9_CDCA_noT	SQ259	Yes	I no T	0.54	0.56	2.53	0.77	2.84	0.56
S9_noT	SQ259	No	I no T	0.40	0.45	5.53	0.99	10.28	0.34

The cells will be further abbreviated as presented in the first column of Table 1. The following short symbols are used for dyes: **D** (D35), **N** (N719), **S8** (SQ258) and **S9** (SQ259). For the 1:n mixtures, the symbol **_n** is included in the abbreviation. Moreover, adding CDCA (symbol **_CDCA**), using iodide electrolyte without TBP (symbol **_noT**) and cobalt-based electrolyte (symbol **_Co**) is also indicated in the abbreviation.

First, we studied the cells filled with typical iodide electrolyte of the same composition as in the previous report for SQ258 and SQ259 (0.1 mM Li^+^ and 0.5 mM TBP)^32^. In most of the cases (Table 1) adding CDCA to squaraine dyes results in lower J_sc_ (since for thinner films N_ph_ is not sufficient) but higher V_OC_, FF and total APCE which confirms that charge separation efficiency improves when there is lesser dye aggregate formation. Following this, the visible and the NIR dyes in different molar ratios were subjected to photovoltaic characterization and it was found that the solar cells **D:S8_5**, **D:S8_1_CDCA** and **N:S9_1** exhibited the best photovoltaic performance (indicated in bold in Table 1). The cells fabricated using dye cocktails in the optimum molar ratios exhibited the best J_SC_, because the synergistic effect of two dyes co-adsorbed on TiO_2_ allow for absorption of more photons. The dye cocktails also exhibited an improved V_OC_ and total APCE compared to V_OC_ and total APCE of the respective pure squaraine dyes without CDCA. This also confirms that they partially act like de-aggregating agents for SQ258 and SQ259.

However, it can be noted that total APCE values of the DSSCs sensitized with squaraine dyes individually with standard electrolyte are relatively low (below 50%, with or without CDCA, see Table 1). This indicates that there are severe limitations in the charge separation efficiency that can originate e.g. from limited electron injection quantum yield or limited dye regeneration quantum yield. To verify the first possibility, the DSSCs were fabricated using the same dye cocktails and with an alternate electrolyte formulation. This electrolyte excluded TBP and included a higher concentration of Li^+^ (0.6 mM) to assess its impact on the device performance. Such composition results in shifting the TiO_2_ conduction band edge towards more positive potentials vs. NHE (or lower energy vs. vacuum) and it is a common procedure to increase electron injection yield^5,44^. It is especially often used for NIR-sensitive dyes for which the energy excess of the excited state with respect to TiO_2_ conduction band is relatively small, i.e., for dyes having low driving force for electron injection^11,41^. The results shown in Table 1 confirm that indeed the photocurrent was greatly enhanced upon changing the electrolyte. For example, for SQ258 total APCE increased by about two times, from ~ 0.3 with the electrolyte consisting of TBP (**S8**, **S8_CDCA**) to ~ 0.6 with the electrolyte that did not contain TBP (**S8_noT**, **S8_CDCA_noT**, Table 1). The sample **D:S8_5_noT** exhibited the best photocurrent (indicated in bold in Table 1). On the other hand, in the cells without TBP, V_OC_ and FF are always lower than in the corresponding cells with TBP, so the final PCE is often not higher.

Finally, we have also tested the performance of selected configurations in cobalt-based electrolyte with TBP. As can be seen from Table 1, V_OC_ is higher while J_SC_ is lower than in the iodide-based electrolyte of the corresponding configuration. It can be explained by more positive redox potential of the cobalt redox pair with respect to the iodide one (by ~ 0.2 V), which increases its difference with respect to Fermi level (higher voltage) but can decrease the quantum yield of dye regeneration of squaraine dyes. Further comparison of the iodide and cobalt electrolytes with respect to charge separation dynamics will be discussed in the next sections. Most importantly, we have observed the best efficiency for the D35:SQ258 1:5 mixture (**D:S8_5_Co**), which is the highest (~ 3.5%) among all studied systems. It means that cobalt-based electrolyte can be a good alternative for the iodide one in semi-transparent co-sensitized squaraine-based cells. Moreover, cobalt-based electrolyte is slightly less yellow than iodide one in DSSC (Figs. S1C, D and S2), which can be another parameter for tuning to the desired color in semi-transparent cells. The mixture N719:SQ259 has not been tried in cobalt-based electrolyte because N719 dye show low performance with Co redox pair^45^.

The photovoltaic results presented in this section show that co-adsorbing suitable dyes and reducing the TBP content in the electrolyte can significantly improve the efficiency of semi-transparent DSSCs using a squaraine dye-based co-sensitized system. Achieving the best power conversion efficiency (PCE) with a thicker TiO_2_ layer would require detailed optimization of the electrolyte composition and identifying the ideal dye mixture ratio. This process might also involve using a certain amount of CDCA and refining the sensitization procedure. However, these aspects were beyond the scope of our current work. Instead, we focused on understanding the specific mechanisms driving the observed improvements in photocurrent, which are explored in detail in the next two sections.

### IPCE spectra

**Fig. 2:**
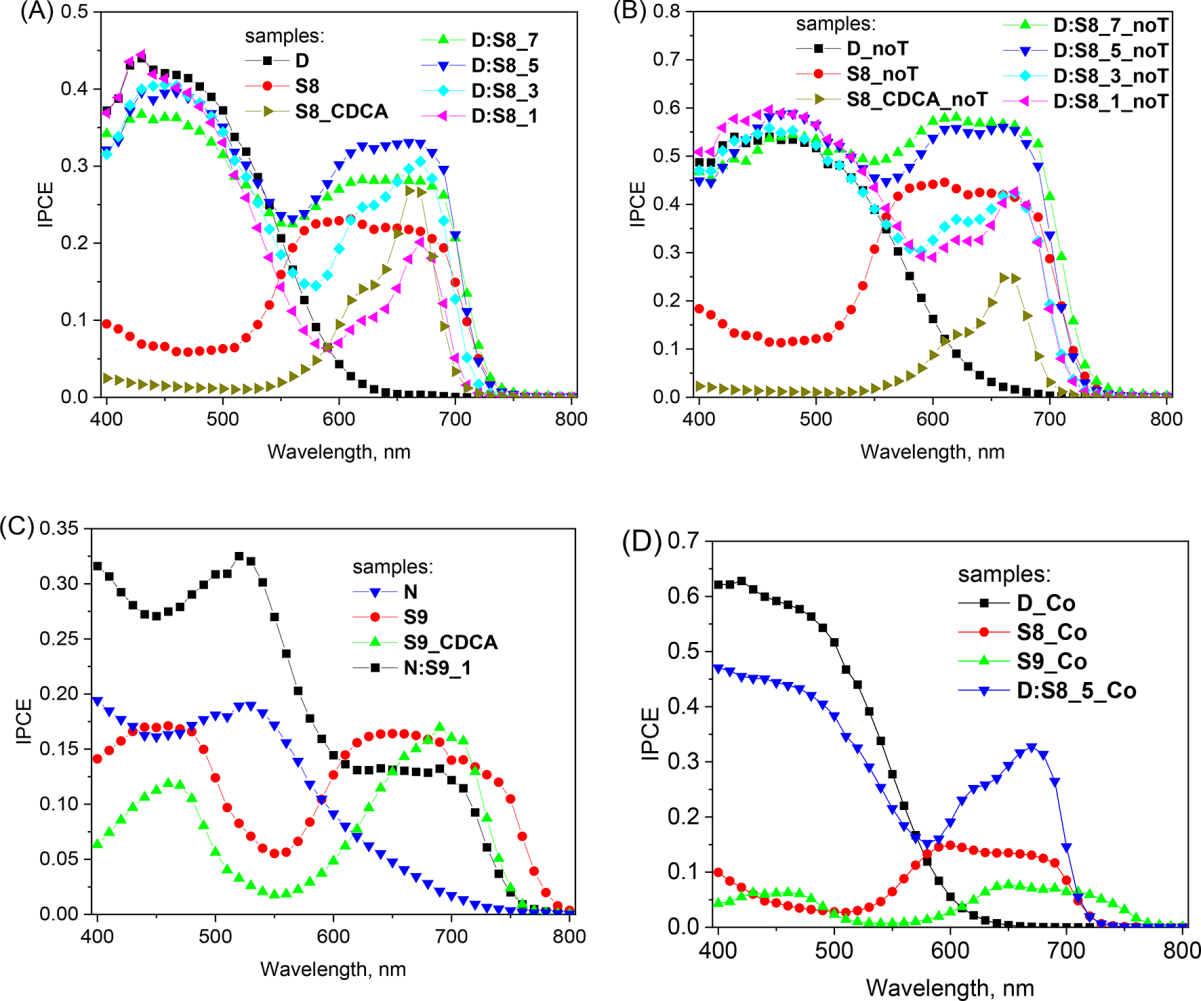
IPCE spectra of cells sensitized with D35 and SQ258 dyes and their mixtures comprising iodide electrolyte containing TBP (**A**) and non-containing TBP (**B**), as well as sensitized with N719 and SQ259 dyes and their mixtures (**C**). Figure (**D**) presents ICPE spectra for selected samples in cobalt electrolyte with TBP.

Figure 2 presents the representative IPCE spectra of the studied systems. Figure 2A shows IPCE for DSSCs prepared with SQ258 and D35 individually and in their different molar ratios along with using iodide electrolyte containing TBP. In Fig. 2B the IPCE spectra for the DSSCs fabricated using the same dyes individually and same dye cocktail ratios are shown but with using iodide electrolyte that did not contain TBP. The IPCE spectra for DSSCs fabricated with SQ259 and N719 individually and their cocktails in different molar ratios are presented in Fig. 2C.

First, it can be observed that the squaraine dye cells without CDCA exhibit a broader response across both the shorter and longer wavelength regions, as indicated by the stationary absorption spectra (section “Stationary absorption”). This observation confirms the significant aggregation of squaraine dyes in the absence of CDCA. Additionally, it suggests that the charge separation efficiency remains consistent across the entire band of aggregates. This conclusion is supported by the similarity between the shape of the IPCE spectra (Fig. 2) and the fraction of absorbed light derived from the absorption spectra (Fig. 1). In principle we could expect that in the cells without CDCA the excitation of monomers (close to the main absorption peak: 660 nm for SQ258 and 700 nm for SQ259) might bring higher IPCE values than direct excitation of aggregates (on both sides of the maximum), as total APCE for cells with CDCA is better than for the cells without CDCA (Table 1). However, as will be shown in the next section, in the aggregated systems there is a fast energy transfer from monomers to aggregates before electron injection takes place. Thus, very likely, in the samples without CDCA most of the electrons are injected from the aggregates, regardless of whether monomer or aggregate is initially excited.

As the addition of D35 or N719 led to prevention of dye-aggregate formation, the IPCE bands for squaraine dyes exhibited significant narrowing after their addition. In the case of D35:SQ258 dye cocktail, gradual narrowing was observed when the molar ratio of D35 was gradually increased from 1:7, 1:5, 1:3 to 1:1 (both with TBP—Fig. 2A and without TBP—Fig. 2B). It should be highlighted that IPCE band of the co-adsorbed N719 or D35 dyes was almost always higher than that of squaraine dyes, while in the stationary absorption their contribution was smaller. It is because in both electrolytes (with and without TBP) total APCE values of N719 and D35 were higher than those of SQ258 and SQ259 (Table 1), which means that their charge separation quantum yield is higher. The only exceptions are IPCE spectra for dye cocktails in the lowest molar ratio and electrolyte without TBP (**D:S8_5_noT** and **D:S8_7_noT**), because in this case the IPCE band of D35 is similar to that of SQ258 (Fig. 2B). Such flat IPCE spectrum over broad spectral range is the desired shape for the co-sensitized DSSC systems optimized for the best PCE.

Finally, Fig. 2D presents IPCE spectra of selected cell in cobalt-based electrolyte. Similar to IPCE spectra in iodide electrolyte, the cells with SQ259 and SQ258 alone exhibit broad features due to aggregation. However, a remarkable result is obtained for 1:5 mixture (sample **D:S8_5_Co**). Not only does this configuration suppress the aggregation of the squaraine dye, but it also results in significantly higher IPCE values within the SQ258 activity range (600–700 nm) compared to SQ258 alone. It indicates increased charge separation quantum yield in co-sensitized system, a finding that will be confirmed in the next section.

For the cells presented in Fig. 2, additional APCE spectra were also calculated and shown in Fig. S3. Generally, the data obtained confirms the total APCE values calculated from J_SC_ of the cells (Table 1). Additionally, APCE spectra in the mixtures can help to distinguish between the charge efficiency of both dyes. In the case of D35:SQ258 mixture it can be observed (Fig. S3) that the APCE values are always higher in the spectral region of D35 (400–500 nm) than SQ258 (600–700 nm). For squaraine dyes in the mixtures an increase in the APCE values with respect to squaraine alone (without CDCA) is clearly visible, and the highest values are for D35:SQ258 1:5 mixture (Fig. S3).

### Transient absorption

Fabricated solar cells (the best devices of each series) were used as samples for transient absorption (TA) measurements. In several cases two or three devices of the same configuration were measured to confirm that the errors in the determined dynamics are relatively small. All the differences in TA features between the different series discussed below are greater than these errors. We have also verified that the contribution of electrolyte to the measured TA spectra is negligible. However, the artifacts from FTO glass and the spectral-dependent broadening of IRF could have some influence on the fastest time components of duration comparable with IRF (0.2 ps or less)^37^. Therefore, these components will not be shown or discussed. For squaraine dyes only (without using co-sensitized dyes) we excited the cells mainly at 475 nm to have good S/N (signal to noise) ratio, and the same excitation wavelength was used for N719:SQ259 composition because the contribution from the TA signal change of N719 is small^36^. However, for D35:SQ258 cells the excitation at 475 nm would result in a complicated TA evolution due to both D35 and SQ258 contribution, so these mixtures were excited at 725 nm, which gives worse S/N ratio but then we do not excite D35 dye at this wavelength. For reference, we have also performed TA studies for both squaraine dyes in ethanol solution (the same which was used for sensitization) excited at 620 nm.

**Fig. 3:**
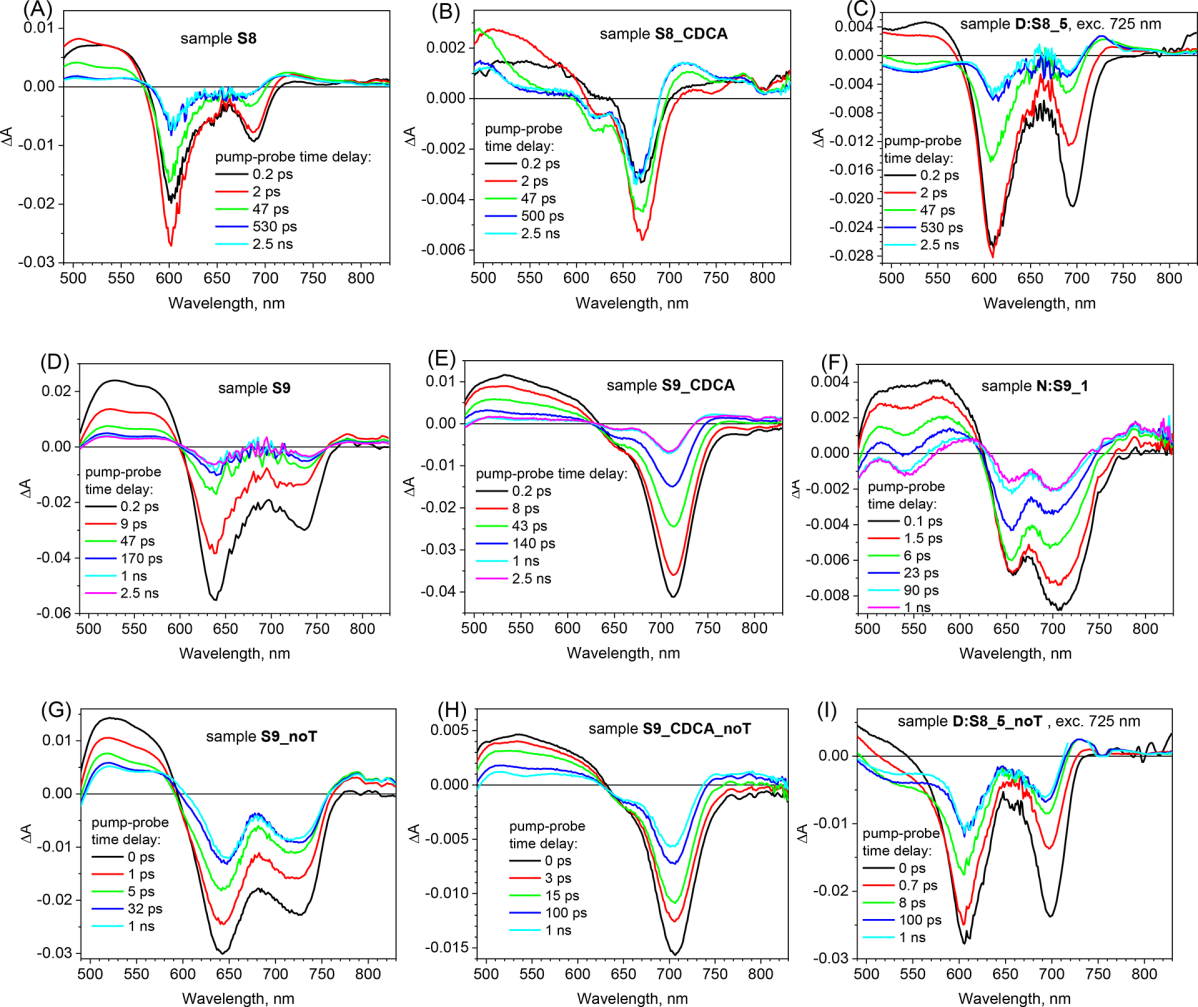
Selected transient absorption spectra of different cells for the indicated times (electrolyte with TBP: **A**–**F**, without TBP: **G**–**I**). The excitation wavelength was 475 nm except for those figures in which it is indicated in figure insets.

Figure 3 presents the exemplary TA spectra for several cells at selected time delays between pump and probe pulse. It should be noticed that for the cells without CDCA the high absorption at the maximum of squaraine bands (around 660 nm for SQ258 and 700 nm for SQ259) results frequently in very low intensity of the probing light and the resultant TA signal at this wavelength is very noisy and coupled with lower amplitude (sometimes even equal to zero). However, it does not influence the analysis and discussion presented below. In Fig. 3 the evolution of TA can be mainly observed as the decay of two TA signals: negative bleach decay in the spectral range of stationary absorption (due to the ground state depopulation) and positive absorption from the excited state of the squaraine dye. The residual signal (constant after 500 ps or less) shows the state of the system after electron injection (oxidized dye absorption and residual ground state depopulation).

First, TA spectra confirm the aggregation effects observed in stationary absorption (section “Stationary absorption”) and IPCE (section “IPCE spectra”) studies. By comparing the samples with and without CDCA, the effect of aggregation on the negative bleach signal is clearly observed: for SQ258 a bleach peak around 670 nm appears with CDCA (sample **S8_CDCA**, Fig. 3B) while without CDCA this bleach shifts to 690 nm and a new peak at 600 nm appears due to aggregation (**S8**, Fig. 3A); for SQ259 these peaks are: 710 nm with CDCA (**S9_CDCA**, Fig. 3E) and 640 and 740 nm without CDCA (**S9**, Fig. 3D). In the mixtures (**D:S8_5**, Fig. 3C and **N:S9_1**, Fig. 3F) the partial effect of de-aggregation can be observed as the ratio of short-wavelength to long-wavelength peaks decrease with respect to the spectra without CDCA. Significant bleach recovery within a short time (tens of ps) indicates that a lot of the excited dye deactivates to the ground state, which could explain the relatively low total APCE values of both squaraine dyes individually (Table 1). The samples with CDCA show slower decay of TA signal and higher relative amplitude of the residual signal (e.g. compare **S8**—Fig. 3A with **S8_CDCA**—Fig. 3B and **S9**—Fig. 3D with **S9_CDCA**—Fig. 3E), in line with increased total APCE. On the contrary, changing the electrolyte from that with TBP to that without TBP results in faster decay of TA signal (due to faster electron injection) but also in higher amplitude of the residual signal (e.g. compare **S9**—Fig. 3D with **S9_noT**—Fig. 3G, **S9_CDCA**—Fig. 3E with **S9_CDCA_noT**—Fig. 3H and **D:S8_5**—Fig. 3C with **D:S8_5_noT**—Fig. 3I) since the electron injection becomes more efficient (in line with total APCE increase in the second electrolyte). Finally, in the samples without CDCA (e.g. Figure 3A) it can be observed that the initial ratio of long-wavelength peak to short-wavelength peak amplitudes quickly decreases at time delay of 2 ps (in favor of short-wavelength peak). It indicates a fast partial energy transfer from monomers to aggregates^38^. Table S2 summarizes the most important TA signals in different spectral ranges and the processes associated with their evolution.

**Fig. 4:**
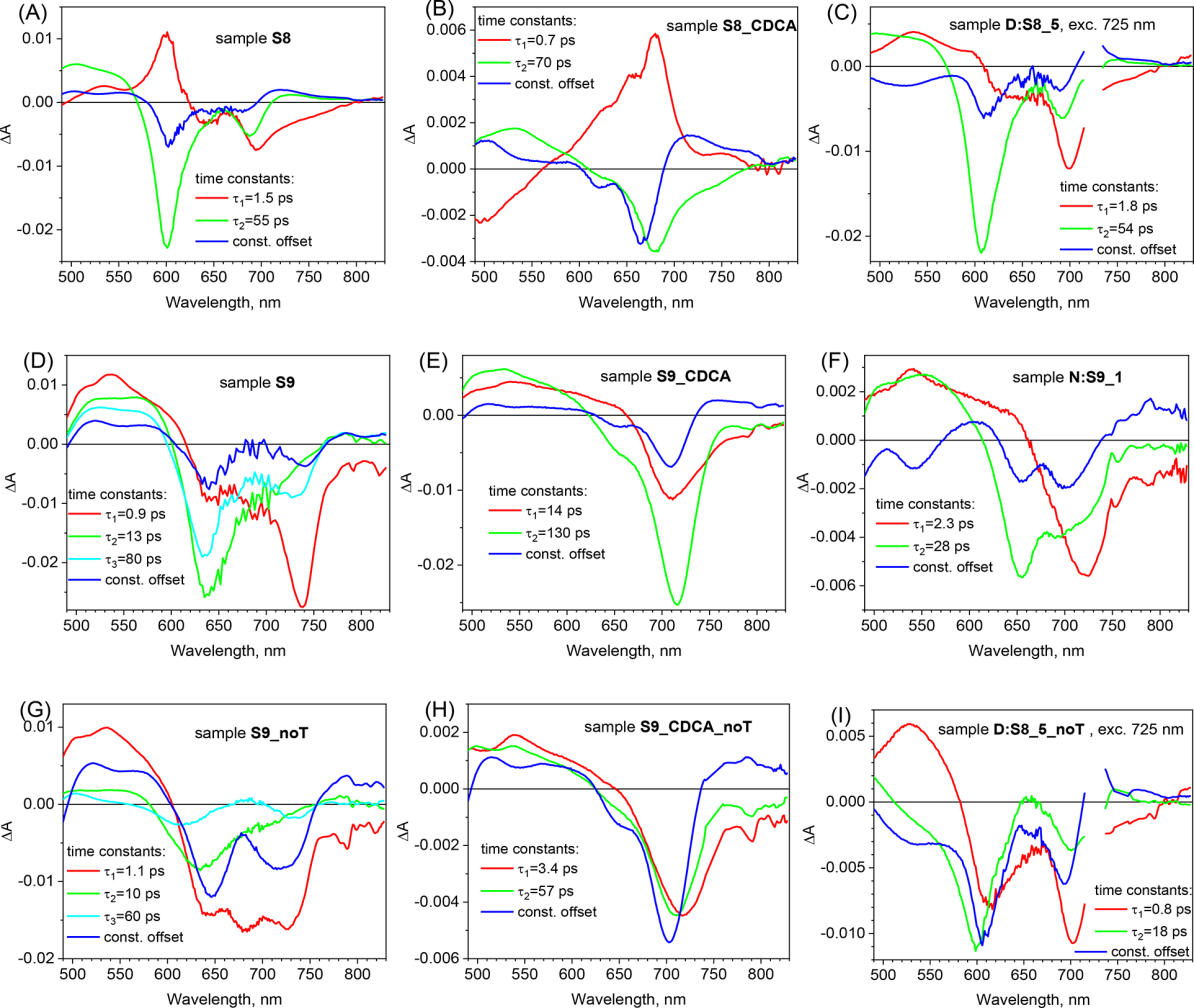
Pre-exponential factor spectra of the indicated time constants obtained from global analysis of TA data of selected cells (electrolyte with TBP: **A**–**F**, without TBP: **G**–**I**), the excitation was at 725 nm 200 nJ in figures (**C**) and (**I**) and at 475 nm, 260 nJ in the rest examples.

To get more insight into TA dynamics of the studied systems, global analysis was performed which gives the characteristic time constants (from multi-exponential fit convoluted with IRF) and the preexponential factor (amplitude) spectra associated with them. Negative amplitude indicates the rise of TA signal while positive amplitude indicates a decay of TA signal. Figure 4 presents the pre-exponential factor spectra of the fitted time constants for selected cells, while the rest of the results for all studied systems is presented in Figs. S4–S6. Two or three exponential fittings with constant offset component was enough to get satisfactory fit quality (an example is shown in Fig. S7). As stated above, the components of time constant equal or below 0.2 ps (IRF) will not be shown and discussed. First, the analysis of TA in solution (Fig. S4) reveals the shape of absorption spectra of the excited state of monomers and their relatively long lifetimes in ethanol (~ 660 ps for SQ258 and ~ 460 ps for SQ259). Components of single or tens of ps of small amplitudes can be related to cooling and solvation processes in the excited states. As the amplitude of the constant offset component added to the fit is practically zero in the whole spectral range, the possible population of the long-living triplet state can be neglected for these squaraine dyes.

**Fig. 5:**
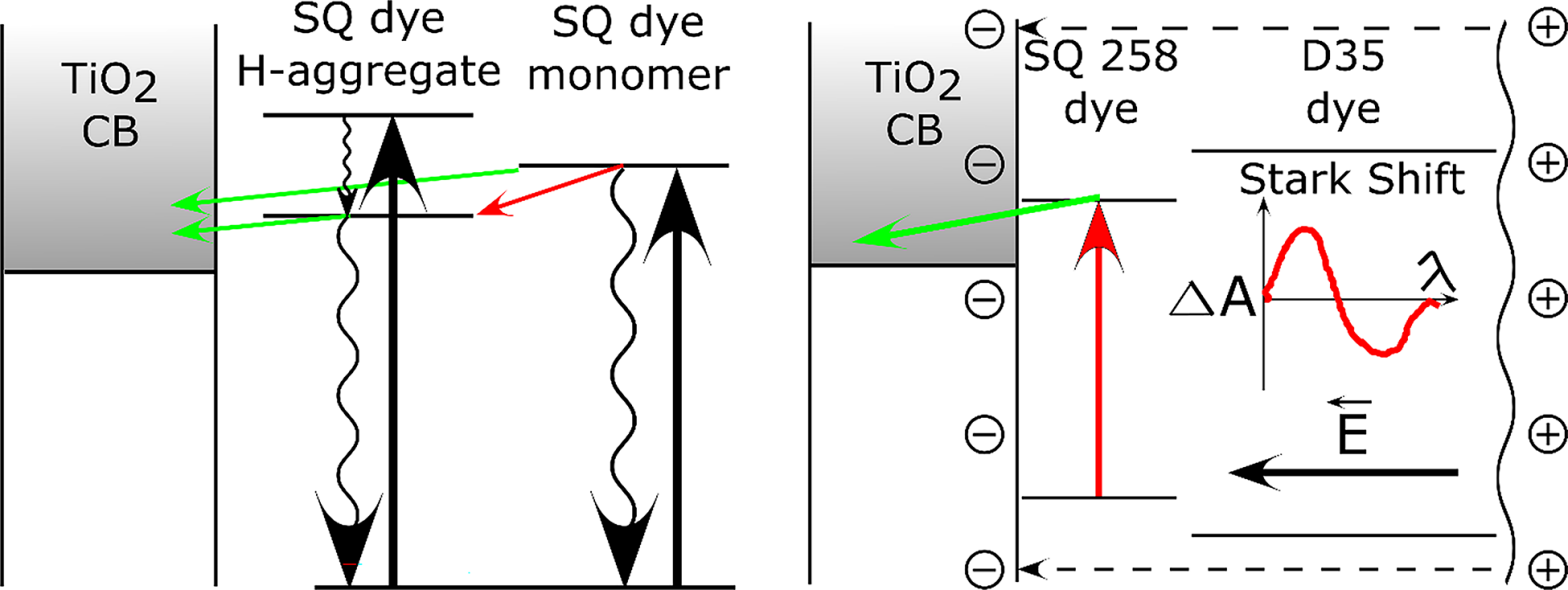
Energetic schemes of the main processes observed in the studied systems: (**A**) electron transfers and internal conversion deactivation in monomers and aggregates, and (**B**) idea of Stark shift effect induced on D35 dye by electrons injected by squaraine dyes.

More interesting and complicated dynamics were observed in the solar cell samples. In one case (sample **S8_CDCA**—Fig. 4B) the fastest component of duration of 0.7 ps is observed which is the mirror image of the lowest excited state spectrum thus it describes the delayed population of this state upon the high energetic excitation at 475 nm. Indeed, for the control experiment with excitation at 725 nm of the same cell this component is absent. Similar components can be observed for other cells (SQ258 without CDCA and SQ259 with and without CDCA) but its duration shortens to that of IRF (0.2 ps), so it is not shown. The next fast time component of duration below 3 ps can be mainly assigned to the above-mentioned partial energy transfer from squaraine monomers to H-aggregates. Indeed, it is observed in the samples without CDCA and its preexponential factor spectra show either a clear bleach recovery of the long-wavelength band (negative amplitude) and a bleach increase of the short-wavelength band (positive amplitude)—see Fig. 4A for **S8**, or more pronounced bleach recovery of the long-wavelength than short-wavelength band—Fig. 4D for **S9**. In the latter case the energy transfer probably overlaps with the fast components of excited state deactivation in the aggregates. At first sight the energy transfer from the state which shows lower energy bleach (smaller bandgap) to that of higher energy bleach (larger bandgap) is counter intuitive. However, as it was shown before by some of us in DSSC^38^, in the case of H-aggregates the excited state is split into a higher exciton level (to which the transition from the ground state is allowed) and a lower exciton level (with forbidden transition from the ground state), which can be populated from the higher exciton level or by energy transfer from the monomer (the mechanism shown in Fig. 5A). Thus, the population of lower exciton level of the aggregate by the energy transfer from monomer excited state decreases the bleach in the monomer and blocks the possible transition from the ground state of the aggregate, so the short-wavelength bleach of the aggregates is enhanced in TA.

Longer time constants of the global fit show the deactivation of the excited state of the squaraine dyes, while the constant offset component—the final transient spectrum of the oxidized dye. The excited state deactivates via the competing processes of internal conversion within the squaraine dye and electron injection to TiO_2_ conduction band. The internal conversion of the dyes in the cells is faster than that observed in solution (550–650 ps) and the electron injection is relatively slower, so the preexponential factor spectra contain significant bleach recovery features (especially in the first electrolyte with TBP). Quite often to get good global fit quality, two exponentials (with similar amplitude spectra) were necessary because electron injection phenomena can be spread over broad time scale and can have different rate constants from the hot and relaxed excited state^35,46^. For example, such two time constants are 14 and 130 ps in Fig. 4E (**S9_CDCA**) and 3.4 and 57 ps in Fig. 4H (**S9_CDCA_noT**). In the samples with CDCA the time decay is on average slower than without CDCA (e.g. compare **S8_CDCA**—Fig. 4B with **S8**—Fig. 4A, **S9_CDCA**—Fig. 4E with **S9**—Fig. 4D or **S9_CDCA_noT**—Fig. 4H with **S9_noT** Fig. 4G) which suggests that the internal conversion is slower in monomers than in aggregates and it works in favor for more efficient electron injection (less blech recovery and higher total APCE). On the other hand, decrease of the time constants in the electrolyte without TBP (e.g. compare **S9_CDCA_noT**—Fig. 4H with **S9_CDCA**—Fig. 4E, **S9_noT**—Fig. 4G with **S9**—Fig. 4D or **S8_noT**—Fig. S6C with **S8**—Fig. 4A) means that electron injection is faster so the quantum yield of this process increases upon shifting TiO_2_ conduction band, residual bleach increases (bleach recovery has smaller amplitude) and total APCE rises.

In principle unwanted bleach recovery observed on ps or ns time scale can be also an indication of the fast electron recombination between TiO_2_ and the oxidized dye^47^. Quite often distinguishing this process from the internal conversion is tough in DSSC samples because TA spectra of the excited state are quite similar to those of the oxidized dye^35^. However, in the present case of squaraine dyes such recombination does not play an important role. It is because the preexponential factor spectra of the decay components show the specific features of the excited state and not the oxidized dye. First, they exhibit characteristic maximum at around 530–540 nm (at least for the monomers—e.g. **S8_CDCA**—Fig. 4B, **S9_CDCA**—Fig. 4E and **S9_CDCA_noT**—Fig. 4H) similar to that of excited state decay component in solution (Fig. S4), while the oxidized dye has a small minimum there (e.g. amplitude of constant offset component in Fig. 4A, B, D, E, G, H). Second, the decay components possess negative amplitude on the long-wavelength part of the stationary absorption (above 720 nm for SQ258 and above 750 nm for SQ259) which is due to the stimulated emission decay of the excited state. It is also observed for the excited state in solution and not for the oxidized dye in cells which has positive amplitude there (both for SQ258 and SQ259). Moreover, the dynamics of the electron recombination between TiO_2_ and the oxidized dye should depend on the pump pulse fluence (faster for higher fluence) because it is a second order process. We have checked in several cases the dependence of the fitted time constants on the pump fluence and observed that it is weak (compare Fig. 4 with Fig. S5). As a conclusion, for the cells with SQ258 and SQ259 the dominating mechanism for the unwanted bleach recovery (and the limitation in total APCE) is the fast internal conversion in the excited state (Fig. 5A).

Global TA analysis in the mixtures once again confirms the de-aggregation function of D35 and N719 dyes on the squaraine dyes as the contribution of short-wavelength aggregation band in the bleach TA signal decreases with respect to the squaraine dye alone (which can be observed in the preexponential factor spectra of both decay components and in the final constant offset component spectra). For example, for D35:SQ258 mixtures the relative contribution of the aggregation bleach at ~ 600 nm gradually decreases from SQ258 alone (Fig. 4A) to 1:5 (Fig. 4C, I), 1:3 (Figs. S6A, S6D) and further to 1:1 ratio (Figs. S6B, S6E). Furthermore, by increasing the contribution of D35 dye in the D35:SQ258 mixture the time constant of SQ258 excited state increases (since its lifetime is longer in monomers than in aggregates). For the electrolyte with TBP this lifetime is 54 ps for 1:5 (Fig. 4C), 70 ps for 1:3 (Fig. S6A) and 160 ps for 1:1 mixture ratio (Fig. S6B); without TBP the corresponding lifetime is shorter (as for squaraine alone) but also increases: from 18 ps for 1:5 (Fig. 4I), 22 ps for 1:3 (Fig. S6D) up to 90 ps for 1:1 mixture ratio (Fig. S6E). Similarly, for N719:SQ259 1:1 mixture the lifetimes of SQ259 excited state are in between those of SQ259 alone without CDCA and with CDCA (Fig. 4D–F).

Next, TA results of D35:SQ258 mixtures reveal another interesting effect: the residual (constant offset component) spectrum shows a negative bleach band in the range 500–550 nm (e.g. Figure 4C, I, and Fig. S6D, E) which is absent in the samples without D35 (e.g. Figure 4A, B and Fig. S6C). This bleach should be assigned to that of D35, however D35 dye cannot be directly excited since in these mixtures 725 nm excitation wavelength was used. In principle, the energy or charge transfer from SQ258 to D35 could occur, but this process is not possible due to the mismatch of the bandgap (D35 has higher bandgap than that of SQ258) and excited state energy (D35 has higher energy than SQ258)^32,48^. Therefore, the only explanation is that the occurrence of the negative signal in 500–550 nm range is due to the Stark shift in stationary absorption of D35 in the electric field at TiO_2_ surface created by the electron injections from SQ258 (see Fig. 5B). The Stark shift in DSSCs is proportional to the derivative of absorption intensity vs. wavelength^49–51^ and indeed, the stationary absorption of D35 is rising in this range (500–550 nm, see e.g. Figure 1A). Moreover, the amplitude of the Stark shift increases with more electrons injected into TiO_2_ (causing higher electric field) and, in line with this property, the negative signal observed by us shows increment when the electron injection from SQ258 is more efficient. It can be noticed by comparing the constant offset spectra, when the electrolyte is switched to that without TBP (giving higher APCE values and thus J_SC_) for the sample with the same mixture ratio: **D:S8_5**—Fig. 4C with **D:S8_5_noTBP**—Fig. 4I, **D:S8_3**—Fig. S6A with **D:S8_3_noT**—Fig. S6D or **D:S8_1**—Fig. S6B with **D:S8_1_noT**—Fig. S6E. Stark shift effect has been frequently reported in TA studies in DSSC^35,49–52^, but according to our knowledge it is the first time when it is observed in co-sensitized system by exciting another dye. The potential application of this “co-Stark shift effect” should be highlighted as it enables direct observation of the electron injection quantum yield from the co-sensitized dye. Stark shift effect related to the same dye from which the electron injection takes place (“self-Stark shift effect”) is often hard to be resolved as it spectrally overlaps with negative bleach band. Moreover, Stark shift signal is present in DSSCs for relatively long time, up to hundreds of µs^49,51^, as it decays due to charge screening by cations present in the electrolyte^53^. Therefore, it is possible to observe it and estimate the electron injection quantum yield by not only using ultrafast TA^52^, but also with other absorption techniques operating at longer time scales, like nanosecond flash photolysis^49,51^ or photoinduced absorption measurements (PIA)^50^.

Finally, Fig. S8 shows global fit results of the selected cells in cobalt-based electrolyte. As can be seen, the time constants for the squaraine dyes alone are similar to those in iodide electrolyte (compare **S9_Co**—Fig. S8A with **S9**—Fig. 4D and Fig. **S8_Co**—S8B with **S8**—Fig. 4A) indicating that the dynamics of the energy transfer from monomers to aggregates and the excited state decay (due to electron injection and internal conversion) does not depend on the electrolyte. However, for the D35:SQ258 mixture (**D:S8_5_Co**—Fig. S8C) the above time constants become longer, as observed in the system with less aggregations. Moreover, the residual constant offset signal at the squaraine bleach band region is much higher, which agrees with huge improvement in total_APCE and IPCE spectra of SQ258 in cobalt-based electrolyte upon co-sensitization. The negative amplitude of the constant offset component in 500–600 nm range due to “co-Stark shift effect” can be also observed (Fig. S8C).

## Conclusions

The co-sensitization of the two squaraine dyes SQ258 and SQ259 with popular visible dyes led to better photovoltaic performance when compared to their individual photovoltaic behavior and show the potential for application in multi-color semi-transparent solar cells. Among the tested dye cocktail ratios, D35 with SQ258 in molar ratio of 1:5 exhibited the highest efficiency of 3.3% in thin-film cells, which is about 70% better than for D35 alone and 130% better than for SQ258 alone. Moreover, switching to cobalt-based electrolyte further enhanced the efficiency of this mixture, raising it to 3.5%. SQ259 also has shown an increase in the performance upon mixing it with N719 dye, in this case the optimum molar ratio of the dye cocktail was found to be 1:1. In addition to enhancing photon harvesting in the visible region, the D35 and N719 dyes used in the dye cocktails also acted as de-aggregating agents, effectively reducing the aggregation of squaraine dyes. This de-aggregating characteristics of D35 and N719 has been proven in stationary absorption, IPCE spectra and transient absorption measurements. The transient absorption studies also showed that fast internal conversion in the excited state of the squaraine dyes attached to TiO_2_ competes with electron injection, thereby limiting the photocurrent. Quantum yield of electron injection of the cells can be improved by modifying the electrolyte composition (more Li^+^ and less TBP) to enhance electron injection rate. Another strategy for improvement is to use CDCA or co-sensitized systems which slows down internal conversion of the excited state since there are more monomers and less energy transfer from monomers to aggregates. Despite lower performance of pure squaraine dyes in cobalt electrolyte, the mixture of dyes results in even better performance than in iodide electrolyte due to improved electron injection quantum yield. Our studies not only provide insights into improving the efficiency of DSSCs with near-infrared dyes but also present important novel findings for understanding dye-dye interactions in co-sensitized systems. These include phenomena such as energy transfer between the monomers and aggregates, mutual de-aggregation function of the co-sensitized dyes and Stark shift effect, which serves as a probe for assessing the electron injection quantum yield of the other dye.

## Electronic supplementary material

Below is the link to the electronic supplementary material.

Supplementary Material 1

## Data Availability

Data is partially provided within the manuscript or supplementary information files, and the rest can be provided upon corresponding author request.
