# Supplementary material for: Investigation on the influence of co-sensitization on semi-transparent DSSCs fabricated using NIR-sensitive squaraine dyes and visible dyes
Source: Sci Rep. 2025 Feb 25;15:6748. doi: 10.1038/s41598-025-90337-0 (PMC11861276; doi:10.1038/s41598-025-90337-0)
Supplement: Supplementary file 1 — Supplementary Material 1 [file 41598_2025_90337_MOESM1_ESM.pdf]

Supporting Information

for

Investigation on the influence of co-sensitization on semi-transparent DSSCs fabricated using NIR-sensitive squaraine dyes and visible dyes

Nur Izyan[1], Adam Glinka[2], Safalmani Pradhan[1], Chinmai Mysorekar[2], Shyam Sudhir Pandey[1] and Marcin Ziółek[2 *]

1 Graduate School of Life Science and Systems Engineering, Kyushu Institute of Technology, 2,4-Hibikino, Wakamatsu, Kitakyushu 808-0196, Japan

2 Faculty of Physics and Astronomy, Adam Mickiewicz University, 2 Uniwersytetu Poznańskiego, 61-614, Poznań, Poland

* corresponding author, e-mail: marziol@amu.edu.pl

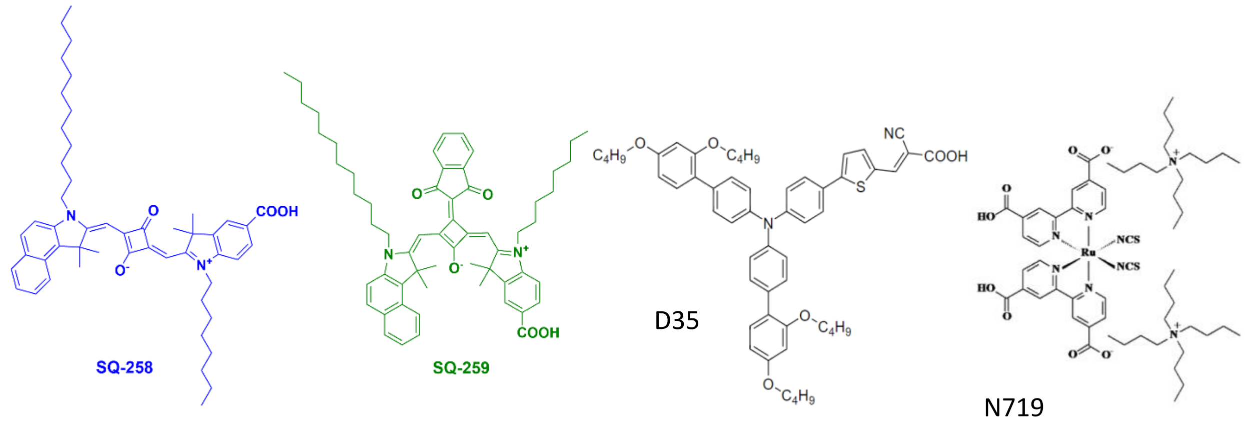

Scheme S1. Molecular structures of the dyes utilized in this research.

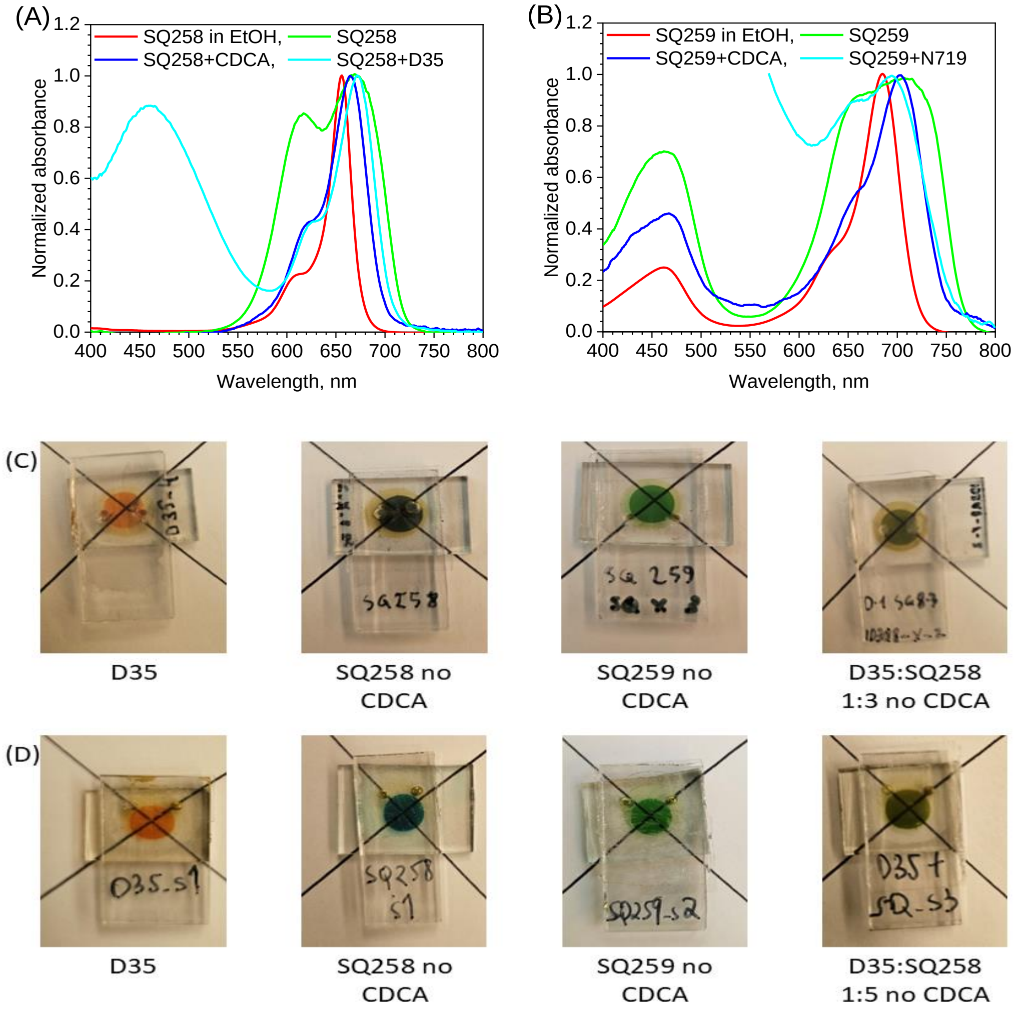

Figure S1. Normalized absorption spectra of squaraine dyes in solution, and on TiO_2: with CDCA, without CDCA and co-sensitized (1:1). (A) is for SQ258 and (B) is for SQ259. Figures (C) and (D) show the photos of different color solar cell samples in iodide and cobalt based electrolyte, respectively. The visibility of the marked X below each cell confirms their semi-transparency.

Table S1. Example of the statistics of the photovoltaic parameters, cells D35:SQ258 (1:1) without CDCA, iodide electrolyte with TBP:

**Cell**	**Voc[V]**	**FF**	**Jsc [mA/cm^2]**	**PCE [%]**	**N_{Ph} [10^{20}*s^{-1}*m^{-2}]**	**Total APCE**
Cell no. 1	0.64	0.67	3.63	1.55	8.68	0.26
Cell no. 2	0.65	0.62	4.14	1.67	8.12	0.32
Cell no. 3	0.69	0.71	3.87	1.90	7.81	0.31
Relative error (std. deviation/mean)	4%	7%	7%	10%	5%	11%

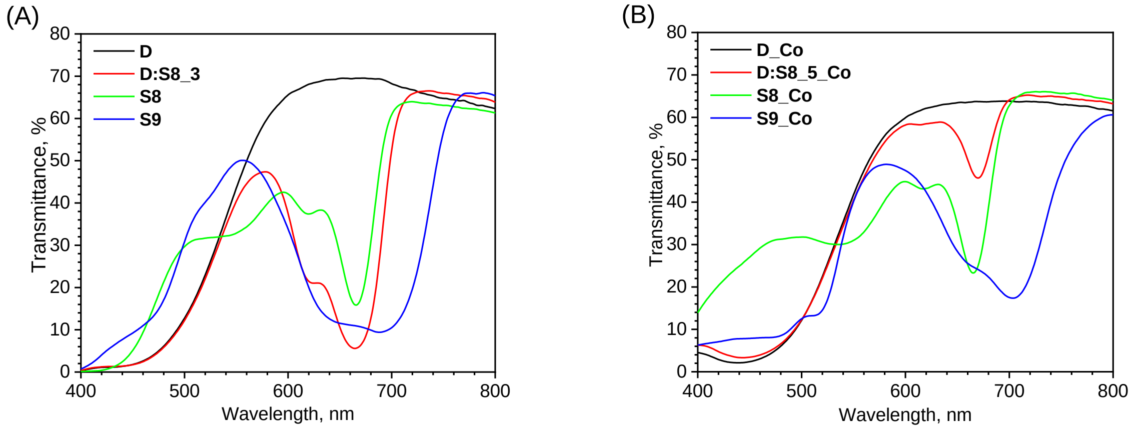

Figure S2. Transmittance spectra of the selected full solar cells without CDCA in iodide (a) and cobalt (b) based electrolytes. The cells are the same as those presented in photos in Figure S1c,d. The cell abbreviations are explained in Table 1 in the main manuscript. It can be noted that in each case, there is at least one part of the spectrum where the transmission is relatively high, above 30%. It can be also noted that in the spectral range below 460 nm the transmission in cobalt-based electrolyte is better than in the iodide electrolyte due to the different absorption of both redox couples.

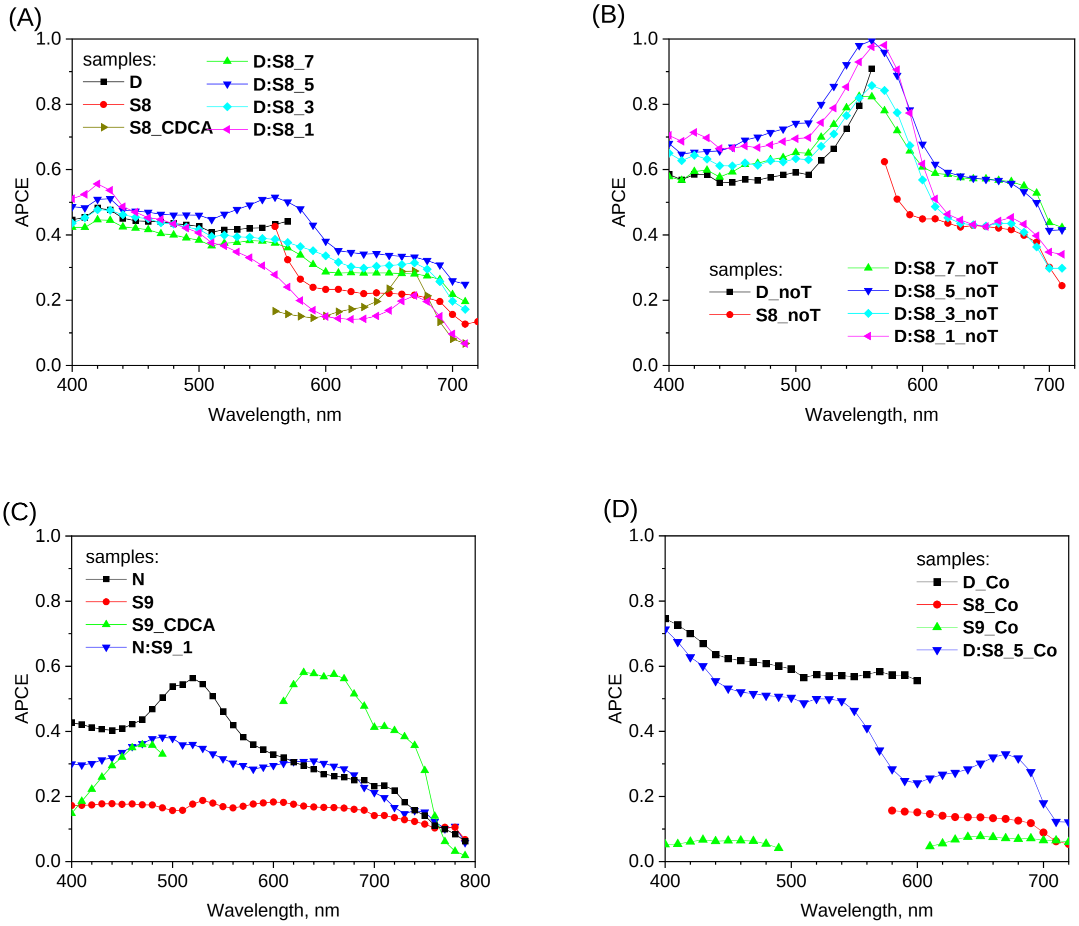

Figure S3. APCE spectra of the selected solar cells, whose IPCE spectra are presented in Figure 2 (in the main manuscript). The cell abbreviations are described in Table 1. APCE values at certain wavelengths were obtained by dividing the IPCE values by light harvesting efficiency calculated from absorption spectra of the electrodes presented in Figure 1 and assuming that the shifts in the absorption due to electrolyte are negligible. Only the spectral ranges at which the absorbance of the electrodes (from Figure 1) is greater than 0.15 are shown as low absorption values can result in high errors in APCE spectra.

Table S2. Characteristic spectral features observed in TA of DSSC with squaraine dyes and their mixtures together with the processes responsible for their evolution:

(a) SQ258, D35:SQ258

**Spectral range**	**Origin**	**TA signal appears due to:**	**TA signal disappears due to:**	**Disappear rate constant depends on:**
500-550 nm	negative signal due to the Stark shift effect of D35 dye	- electron injection from SQ258 to TiO_2	- electron recombination (but not observed on the TA time scale)	-
580-630 nm	negative bleach signal due to H-aggregates of SQ258	- direct excitation of aggregates<br>- energy transfer from monomers	- internal conversion in aggregates	- internal conversion rate in aggregates<br>- electron injection rate from aggregates to TiO_2
650-710 nm	negative bleach signal due to monomers of SQ258	- direct excitation of monomers	- energy transfer to aggregates<br>- internal conversion in monomers	- energy transfer rate to aggregates<br>- internal conversion rate in monomers<br>- electron injection rate from monomers to TiO_2

(b) SQ259, N719:SQ259

**Spectral range**	**Origin**	**TA signal appears due to:**	**TA signal disappears due to:**	**Disappear rate constant depends on:**
610-670 nm	negative bleach signal due to H-aggregates of SQ259	- direct excitation of aggregates<br>- energy transfer from monomers	- internal conversion in aggregates	- internal conversion rate in aggregates<br>- electron injection rate from aggregates to TiO_2
690-750 nm	negative bleach signal due to monomers of SQ259	- direct excitation of monomers	- energy transfer to aggregates<br>- internal conversion in monomers	- energy transfer rate to aggregates<br>- internal conversion rate in monomers<br>- electron injection rate from monomers to TiO_2

(c) graphical visualization of the spectral features for D35:SQ258 (left) and N719:SQ259 (right):

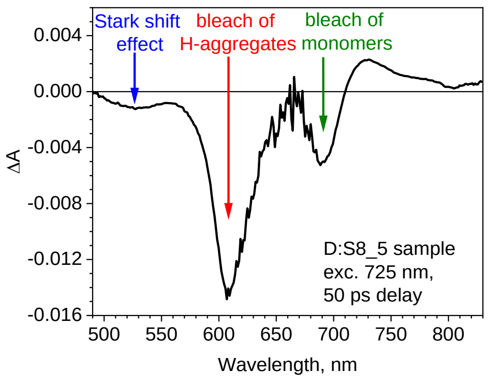

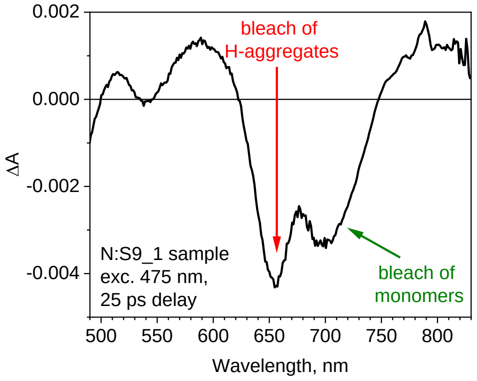

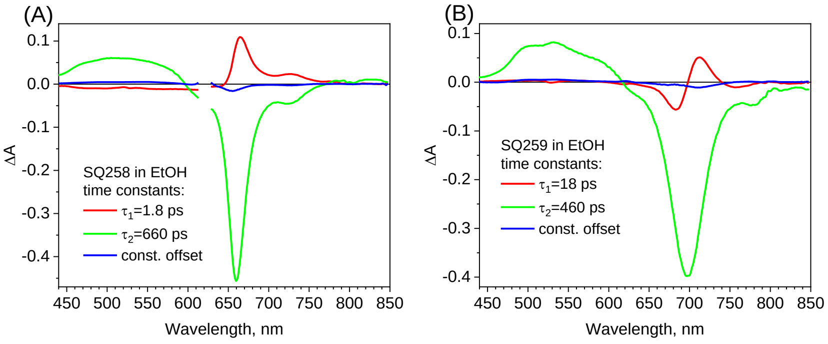

Figure S4. Pre-exponential factor spectra of the indicated time constants obtained from global analysis of TA data of both squaraine dye in ethanol solution, excitation at 620 nm, 600 nJ.

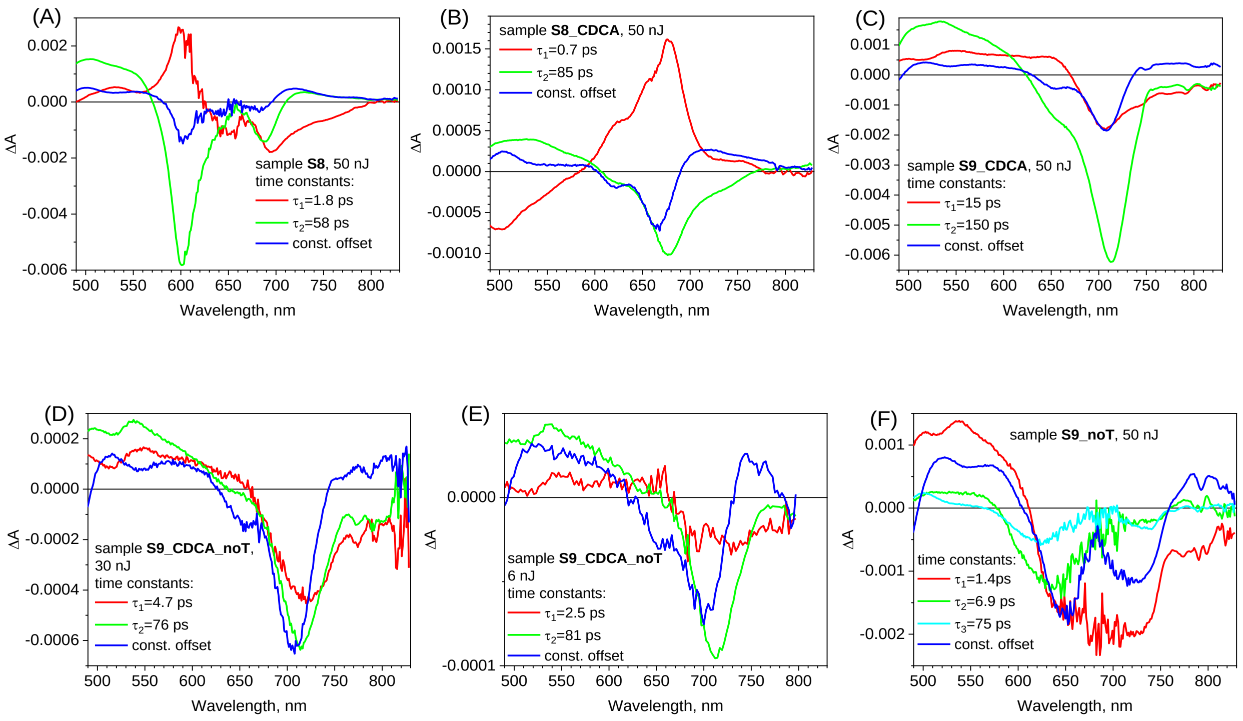

Figure S5. Pre-exponential factor spectra of the indicated time constants obtained from global analysis of TA data of selected cells at low pump fluence, the excitation was at 475 nm.

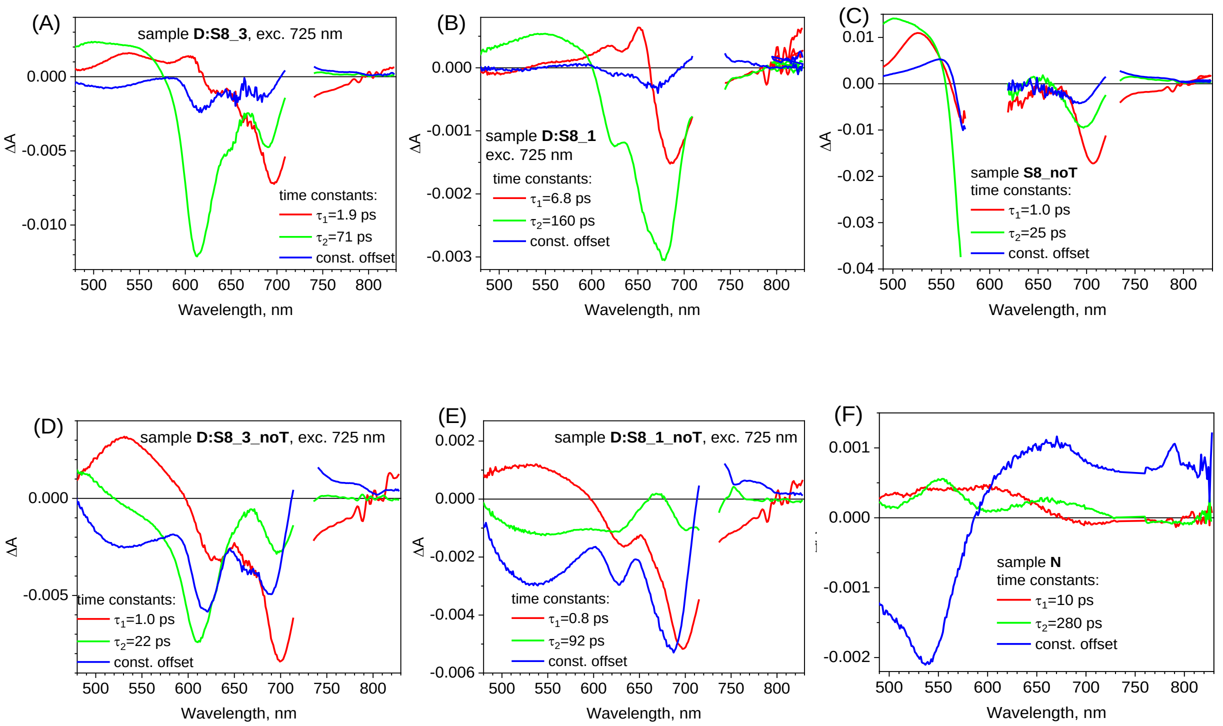

Figure S6. Pre-exponential factor spectra of the indicated time constants obtained from global analysis of TA data of the cells with 1:1 and 1:3 D35:SQ258 mixtures, the excitation was at 725 nm, 200 nJ (A, B, D, E). Additionally, the results for the cell of SQ258 without TBP (C) and of N719 with TBP (F, excitation at 475 nm, 260 nJ) are also presented.

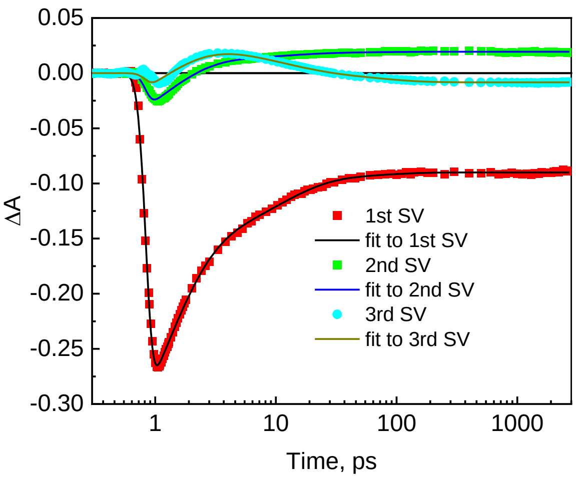

Figure S7. Example of global 3-exponential fit quality of the data presented in Figure 4G – kinetic fits to the first three most significant singular values (SV). The time scale is shown in the logarithmic scale so the time zero is shifted to 1 ps.

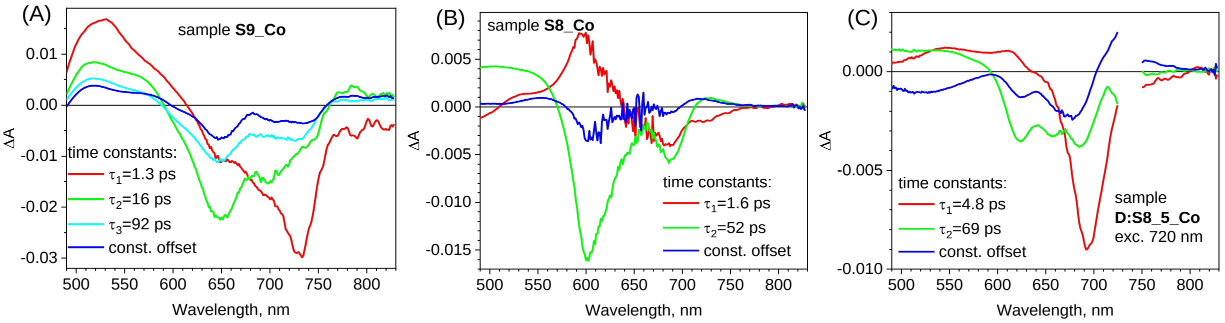

Figure S8. Pre-exponential factor spectra of the indicated time constants obtained from global analysis of TA data of the selected cells cobalt based electrolyte. The excitation was at 475 nm, 260 nJ for (A) and (B) and 720 nm, 200 nJ for (C).
